# Systematic analysis of genetic variants in Han Chinese patients with sporadic Parkinson’s disease

**DOI:** 10.1038/srep33850

**Published:** 2016-09-22

**Authors:** Lamei Yuan, Zhi Song, Xiong Deng, Wen Zheng, Yi Guo, Zhijian Yang, Hao Deng

**Affiliations:** 1 Center for Experimental Medicine, the Third Xiangya Hospital, Central South University, Changsha, China; 2 Department of Neurology, the Third Xiangya Hospital, Central South University, Changsha, China; 3 Department of Medical Information, Information Security and Big Data Research Institute, Central South University, Changsha, China

## Abstract

Parkinson’s disease (PD) is one of the most common neurodegenerative disorders. Accumulated evidence confirms that genetic factors play a considerable role in PD pathogenesis. To examine whether point variants or haplotypes are associated with PD development, genotyping of 35 variants in 22 PD-related genes was performed in a well-characterized cohort of 512 Han Chinese PD patients and 512 normal controls. Both Pearson’s χ^2^ test and haplotype analysis were used to evaluate whether variants or their haplotypes were associated with PD in this cohort. The only statistically significant differences in genotypic and allelic frequencies between the patients and the controls were in the DnaJ heat shock protein family (Hsp40) member C10 gene (*DNAJC10*) variant rs13414223 (*P* = 0.004 and 0.002, respectively; odds ratio = 0.652, 95% confidence interval: 0.496–0.857). No other variants or haplotypes exhibited any significant differences between these two groups (all corrected *P* > 0.05). Our findings indicate that the variant rs13414223 in the *DNAJC10* gene, a paralog of PD-related genes *DNAJC6* and *DNAJC13*, may play a protective role in PD. This suggests it may be a PD-associated gene.

Parkinson’s disease (PD, MIM 168600) is the second most common progressive age-related neurodegenerative disorder affecting approximately 1–2% of the population over 65^1^,^2^. Autopsy studies show a selective loss of dopaminergic neurons in the midbrain substantia nigra, and Lewy bodies formation accompanied by alpha-synuclein aggregation^3^,^4^. Motor skills are primarily devastated by the preferential demise of dopaminergic neurons. A variety of non-motor symptoms were also observed in most PD patients, which may be progressive and precede motor deficits^5^,^6^,^7^,^8^,^9^. Bradykinesia, asymmetric rest tremor, rigidity, and postural instability are the most significant motor symptoms of PD^8^,^10^. Although the etiology remains both complex and elusive, PD is currently acknowledged to be a multifactorial disorder related to genetic factors, aging, environmental exposures, epigenetic factors, and their synergistic interaction^1^,^4^,^11^,^12^. Genetic factors acting as disease-causing determinants, risk or protective factors, can contribute considerably to PD pathogenesis^4^,^7^,^13^. Over the last two decades, at least 23 genetic loci (*PARK1* to *PARK23*), and 18 disease-associated genes, have been implicated in familial and sporadic PD^14^,^15^,^16^,^17^. Approximately 10% of PD cases report a positive family history with the vast majority of cases having undefined genetic causes^4^,^7^,^18^. A polygenic model has been proposed to explain the genetic role in PD pathogenesis^13^. Even though the exact pathogenic mechanisms remain unclear, a complex and synergistic set of mitochondrial defects, and cellular processes including oxidative stress, lysosomal dysfunction, and vesicle trafficking are suggested to have a central role in PD pathogenesis^4^,^8^,^17^. Disease-modifying or neuroprotective therapies, targeting specific pathogenesis to slow or halt progression, are urgently needed as current dopamine replacement therapies only provide symptomatic relief^5^,^9^,^18^. Accumulating evidence suggests that genetic variants may exert a risk or protective role in PD, while results from other studies are inconclusive or inconsistent, and are not replicable^14^,^19^,^20^,^21^,^22^. This study aimed to investigate genetic and allelic frequencies of point variants in a large cohort of 512 Han Chinese PD patients and 512 ethnicity-matched healthy controls, and to evaluate whether the variants or haplotypes are associated with PD development.

## Results

All 35 variants in the 22 potentially PD-associated genes enrolled in this study were examined in the 512 PD patients and 512 healthy controls with perfectly designed primers (see Supplementary Table S1). No departure from Hardy-Weinberg equilibrium for the enrolled variants was observed (all *P* > 0.05). No alterative genotypes, i.e., monomorphisms, were observed in the following 21 variants: rs60003608, rs10935014, rs79953286, rs2227851, rs34086109, rs34322892, rs34845648, rs11538692, rs77570025, rs3752321, rs3764740, rs34594498, rs74942016, rs61744200, rs11570680, rs375681722, rs538881762, the recently reported p.T1367N variant in the teneurin transmembrane protein 4 gene (*TENM4*), rs35693565, rs62444122, and rs199910950. Table 1 shows genotypic and allelic frequencies for variants with two or three genotypes in PD patients and controls. Associations between the variants and PD were assessed. After Bonferroni correction, statistically significant difference between the PD patients and control groups was only observed in the genotypic distribution of the DnaJ heat shock protein family (Hsp40) member C10 gene (*DNAJC10*) variant rs13414223 (χ^2^ = 11.109, *P* = 0.004, corrected *P* = 0.012). The patient group had significantly lower frequencies of the A allele (χ^2^ = 9.523, *P* = 0.002, corrected *P* = 0.004, odds ratio = 0.652, 95% confidence interval: 0.496–0.857) compared to the control group. No statistically significant differences in genotypic or allelic frequencies between the two groups were found in the other 13 variants (all corrected *P* > 0.05, Table 1). No potential PD-association was identified (all *P* > 0.05, Table 2) for haplotypes of enrolled variants, rs6788448-rs35424709 (*ATP13A4*), rs1721100-rs1989754 (*FGF20*), rs33949390-rs34410987 (*LRRK2*), rs3758549-rs4919621 (*PITX3*), and rs2076485-rs7757931 (*UBD*).

## Discussion

The present study investigated possible associations between the 35 variants and PD development in a well-defined cohort of Han Chinese patients with PD. As previously noted, the association between gene variants and the presence or severity of PD is inconclusive and inconsistent either in the same or different populations^3^,^14^,^20^,^21^. Three DNAJ family genes have been implicated in familial neurodegenerative disorders, including the *DNAJC6* gene in autosomal recessive PD (PARK19), the *DNAJC13* gene in autosomal dominant late-onset PD (PARK21), and the *DNAJC5* gene in autosomal dominant adult-onset neuronal ceroid lipofuscinosis^22^,^23^,^24^,^25^,^26^. This study found that only *DNAJC10* gene variant rs13414223 was related to decreased PD risk.

The *DNAJC10* gene (MIM 607987), which is mapped to chromosome 2q32.1, contains 24 exons and spans ~64 kb, is a paralog of two known PD-related genes, *DNAJC6* and *DNAJC13*^23^,^25^,^27^. It encodes a ~91 kDa endoplasmic reticulum (ER) co-chaperone with 793 amino acids, also known as ERdj5, or JPDI, which is a type III DnaJ protein. It is an ER-resident molecule composed of an N-terminal hydrophobic sequence, a type III DnaJ domain, four thioredoxin-like domains, and a C-terminal tetrapeptide KDEL motif mediating ER retention^27^,^28^. It is ubiquitously expressed, ER-localized, and particularly abundant in secretory cells. It is present in the central nervous system, with strong signals in the hippocampus and the granular cell layer of the cerebellar cortex, and moderate signals in the striatum, hypothalamus, and brain stem^27^. The ER-resident luminal protein, DNAJC10, probably acts as a DnaJ-like partner of BiP (immunoglobulin heavy chain-binding protein), and interacts with the ER-resident chaperone BiP through the DnaJ domain in an ATP-dependent manner, which may be up-regulated upon ER stress^27^,^28^,^29^. It is a member of a supramolecular ER-associated degradation complex, recognizing and unfolding misfolded proteins for efficient retrotranslocation^29^. Its reductase activity can split incorrect disulfide bonds in misfolded proteins and facilitate misfolded proteins solubility and ER-associated degradation through its physical and functional associations with the ER degradation-enhancing alpha-mannosidase-like protein and by modulating BiP activity^29^,^30^,^31^,^32^.

PD is a multifactorial disorder attributed to misfolded protein accumulation or aggregates, such as alpha-synuclein, within the ER lumen modulating ER stress and impairing mitochondrial functioning, and referring to neuron degeneration^2^,^5^,^7^. ER contributes to protein quality control and maintaining normal protein function^33^. ER stress, a salient signature of PD, leads to accumulation of ER-associated degradation substrates, generation of reactive oxygen species which contributes to oxidative stress and an inflammatory response, and mitochondrial dysfunction. It then causes neuronal cell death and is responsible for neurodegeneration^34^,^35^. Three *DNAJC10* paralogous genes contribute to familial neurodegenerative disorders via different mechanisms. Impaired synaptic vesicle recycling and perturbed clathrin-mediated endocytosis related to loss-of-function mutations have been reported in autosomal recessive *DNAJC6*-PD^23^,^24^. Toxic gain-of-function and impaired endosomal transport were observed in autosomal dominant PD patients with the *DNAJC13* mutation^25^. In addition, the dominant negative effect of *DNAJC5* mutations leading to presynaptic dysfunction and lysosomal accumulation of misfolded proteins may cause neurodegeneration^26^. DNAJC10 is expressed in the cortex, striatum, hypothalamus, and brain stem, which are sites of neuron degeneration and Lewy body deposition in PD patient brains^9^,^27^. ER luminal protein dnj-27, a mammalian DNAJC10 ortholog, showed a protective role against PD, Alzheimer and Huntington diseases in transgenic Caenorhabditis elegans models. As an age-related proteotoxicity regulator, it exerts a protective function by altering cytoplasmic protein homeostasis and mitochondrial fragmentation caused by alpha-synuclein, beta-amyloid, and polyglutamine peptides^36^. This is consistent with the hypothesized association between PD and the *DNAJC10* gene.

In this study, the variant rs13414223 in the *DNAJC10* gene had a protective role against PD development. Given that this study did not cover either single nucleotide polymorphisms (SNPs) with a minor allele frequency of less than 5% or non-single base substitution variants, other genetic variants such as low-frequency variants, complex variants, non-coding variants involving in the genetic or epigenetic regulatory region, and synergistic or antagonistic effects should be further investigated to evaluate their roles in PD development in Han Chinese populations^1^,^9^,^37^,^38^,^39^.

In summary, the variant rs13414223 in the *DNAJC10* gene may exert a protective role against PD in Han Chinese. This is the first effort, to our knowledge, to explore potential associations between a *DNAJC10* gene variant and PD. Further research which should include a functional study and confirmation in larger patient cohorts of other ethnicities is warranted. These findings may lead to a more complete comprehension of PD pathogenesis and result in personalized and targeted disease-modifying PD therapeutics.

## Methods

### Study participants and clinical evaluation

In this study, a total of 1,024 unrelated Han Chinese individuals from mainland China were enrolled between December 2007 and August 2015. The participants included 512 patients with sporadic PD and 512 matched normal controls (male/female: 308/204) considering age, gender, race, and geographic origin. Patients were recruited through the Department of Neurology, the Third Xiangya Hospital of Central South University, Changsha, China. PD diagnoses were clinically made by two independent neurologists according to a published diagnostic basis. Secondary parkinsonism caused by other known reasons was eliminated^8^,^40^. The ages of patients and controls were 65.8 ± 10.3 years and 65.9 ± 10.5 years, respectively. In patients, the age at symptom onset was 62.4 ± 7.8 years. Some of the recruited PD cases had been previously screened for mutations in the PD-associated genes that were suspected of causing their symptoms. Of the patients, 25.39% (130/512) and 66.21% (339/512) had no mutation in the VPS35, retromer complex component gene (*VPS35*) or the F-box protein 48 gene (*FBXO48*) respectively. Another, 74.80% (383/512) were negative for mutations in either the S100 calcium binding protein B gene (*S100B*) or the RAB39B, member RAS oncogene family gene (*RAB39B*). Of those tested, 59.77% (306/512) and 97.66% (500/512) were negative for point mutations (p.A502V and p.R1205H) in the eukaryotic translation initiation factor 4 gamma 1 gene (*EIF4G1*), and variants (rs10788972 and rs12046178) in the transcription elongation factor A N-terminal and central domain containing 2 gene (*TCEANC2*). All patients were genotyped for seven SNPs to explore any association between variants and PD risk. These included three variants (rs3212366, rs33932559, and rs34090186) in the melanocortin 1 receptor gene (*MC1R*), two variants (rs75932628 and rs2234253) in the triggering receptor expressed on myeloid cells 2 gene (*TREM2*), and two variants (rs1801131 and rs1801133) in the methylenetetrahydrofolate reductase gene (*MTHFR*)^3^,^14^,^40^,^41^,^42^. Normal control subjects were healthy volunteers and denied either a personal or a family history of PD in consanguineous relatives. They were free of other related neurological disorders when examined^12^,^14^. The study was approved by the Institutional Review Board of the Third Xiangya Hospital, Central South University, which follows the Declaration of Helsinki guidelines. Written informed consent was obtained from all subjects from whom peripheral venous blood was drawn to extract genomic DNA. The methods were carried out in accordance with the approved guidelines.

### Selection of variants

The following criteria were used to select the variants enrolled in this study: previously reported variants that confer a PD risk in some populations, and variants meeting certain conditions for the potential PD candidate genes, which include known PD-causing genes, reported PD-related genes, and their paralogs. Point variants in candidate genes have minor allele frequencies higher than 5%, particularly in Asian or Han Chinese populations. Variants are referred to by their reference SNP ID numbers (rs#) as recorded in the database of SNPs (http://www.ncbi.nlm.nih.gov/SNP/)^43^. Prediction results using bioinformatics analysis programs, Sorting Intolerant from Tolerant (http://sift.jcvi.org/), Polymorphism Phenotyping version 2 (http://genetics.bwh.harvard.edu/pph2/), or MutationTaster (http://www.mutationtaster.org/), support the potential deleterious or disease-causing effect of variants^44^,^45^,^46^.

### DNA extraction and variant genotyping

Genomic DNA was isolated from peripheral blood using standard protocols for genetic analysis^40^. Variants genotyping was done using matrix-assisted laser desorption/ionization time-of-flight mass spectrometry by Bioyong Technologies (Beijing, China) following manufacturers’ instructions^14^,^47^. Locus-specific amplifying primers, and single-base extending primers, were designed using Sequenom Assay Design 3.1 software, and were synthesized and diluted as required. Primer quality was assayed using a mass spectrometric system^42^,^48^. Locus-specific amplification by multiplex PCR, and purification of PCR products, were conducted as previously described^14^,^47^,^49^. MassARRAY Typer 4.0 software (Sequenom) was used to analyze spectrometric results and generate the genotype data of each variant^50^. All the procedures were performed by investigators blinded to sample status, i.e., from case or control subjects. Duplicate samples, positive and negative controls, were included to confirm genotyping accuracy. Direct sequencing of the amplicons containing these variants in 8% of randomly selected samples was carried out as quality controls to test the reliability^51^,^52^.

### Statistical analysis

Statistical analysis was performed using Predictive Analytics Software Statistics 18 (SPSS, Chicago, IL, USA). Hardy-Weinberg equilibrium was evaluated to test for the presence of deviation from normal heterogeneity^14^,^42^. Pearson’s χ^2^ test was used to analyze genotype and allele distribution. Haplotype construction and genetic association analysis were performed using SHEsis Online Version (http://analysis.bio-x.cn) following the instructions^53^,^54^. *P* values, odds ratios, and 95% confidence intervals were estimated for statistical results. All statistical tests were two-sided, and *P*-value standing for statistical significance was set at lower than 0.05, as described in previous studies^14^,^48^.

## Additional Information

**How to cite this article**: Yuan, L. *et al*. Systematic analysis of genetic variants in Han Chinese patients with sporadic Parkinson’s disease. *Sci. Rep.* **6**, 33850; doi: 10.1038/srep33850 (2016).

## Supplementary Material

Supplementary Information

## Figures and Tables

**Table 1:** Genotypic and allelic distributions of variants in the PD-related genes in PD patients and controls.

dbSNP ID	Gene^a^	Genotype/Allele	Patients^b^	Controls^b^	χ^2^ value	*P* value	OR (95% CI)
rs6788448	*ATP13A4*	TT	180 (0.352)	158 (0.309)			
		TC	237 (0.463)	269 (0.525)			
		CC	95 (0.185)	85 (0.166)	4.011	0.135	
		T	597 (0.583)	585 (0.571)			
		C	427 (0.417)	439 (0.429)	0.288	0.591	0.953 (0.800–1.136)
rs35424709	*ATP13A4*	TT	455 (0.889)	458 (0.895)			
		TA	57 (0.111)	54 (0.105)			
		AA	0 (0)	0 (0)	0.091	0.763	1.063 (0.716–1.576)
		T	967 (0.944)	970 (0.947)			
		A	57 (0.056)	54 (0.053)	0.086	0.770	1.059 (0.722–1.553)
rs6350	*SLC6A3*	CC	498 (0.973)	494 (0.965)			
		CT	14 (0.027)	18 (0.035)			
		TT	0 (0)	0 (0)	0.516	0.472	0.772 (0.380–1.568)
		C	1010 (0.986)	1006 (0.982)			
		T	14 (0.014)	18 (0.018)	0.508	0.476	0.775 (0.383–1.566)
rs13414223	*DNAJC10*	CC	420 (0.820)	376 (0.734)			
		CA	86 (0.168)	129 (0.252)			
		AA	6 (0.012)	7 (0.014)	11.109	**0.004**	
		C	926 (0.904)	881 (0.860)			
		A	98 (0.096)	143 (0.140)	9.523	**0.002**	0.652 (0.496–0.857)
rs1721100	*FGF20*	GG	127 (0.248)	132 (0.258)			
		GC	262 (0.512)	260 (0.508)			
		CC	123 (0.240)	120 (0.234)	0.141	0.932	
		G	516 (0.504)	524 (0.512)			
		C	508 (0.496)	500 (0.488)	0.125	0.724	0.969 (0.815–1.153)
rs1989754	*FGF20*	CC	129 (0.252)	126 (0.246)			
		CG	262 (0.512)	263 (0.514)			
		GG	121 (0.236)	123 (0.240)	0.054	0.974	
		C	520 (0.508)	515 (0.503)			
		G	504 (0.492)	509 (0.497)	0.049	0.825	1.020 (0.858–1.213)
rs2924835	*LRRK1*	GG	189 (0.369)	215 (0.420)			
		GA	245 (0.479)	238 (0.465)			
		AA	78 (0.152)	59 (0.115)	4.410	0.110	
		G	623 (0.608)	668 (0.652)			
		A	401 (0.392)	356 (0.348)	4.244	0.039	1.208 (1.009–1.446)
rs33949390	*LRRK2*	GG	485 (0.947)	496 (0.969)			
		GC	27 (0.053)	16 (0.031)			
		CC	0 (0)	0 (0)	2.937	0.087	1.726 (0.918–3.243)
		G	997 (0.974)	1008 (0.984)			
		C	27 (0.026)	16 (0.016)	2.874	0.090	1.706 (0.914–3.186)
rs34410987	*LRRK2*	CC	503 (0.982)	510 (0.996)			
		CT	9 (0.018)	2 (0.004)			
		TT	0 (0)	0 (0)	4.503	0.034	0.219 (0.047–1.019)
		C	1015 (0.991)	1022 (0.998)			
		T	9 (0.009)	2 (0.002)	4.479	0.034	0.221 (0.048–1.024)
rs3758549	*PITX3*	CC	370 (0.723)	346 (0.676)			
		CT	130 (0.254)	145 (0.283)			
		TT	12 (0.023)	21 (0.041)	4.077	0.130	
		C	870 (0.850)	837 (0.817)			
		T	154 (0.150)	187 (0.183)	3.832	0.050	0.792 (0.627–1.000)
rs4919621	*PITX3*	TT	295 (0.576)	303 (0.592)			
		TA	181 (0.354)	189 (0.369)			
		AA	36 (0.070)	20 (0.039)	4.851	0.088	
		T	771 (0.753)	795 (0.776)			
		A	253 (0.247)	229 (0.224)	1.563	0.211	1.139 (0.929–1.398)
rs2254562	*SYNJ1*	TT	197 (0.385)	202 (0.394)			
		TC	243 (0.474)	238 (0.465)			
		CC	72 (0.141)	72 (0.141)	0.115	0.944	
		T	637 (0.622)	642 (0.627)			
		C	387 (0.378)	382 (0.373)	0.052	0.820	1.021 (0.854–1.221)
rs2076485	*UBD*	TT	320 (0.625)	316 (0.617)			
		TC	156 (0.305)	176 (0.344)			
		CC	36 (0.070)	20 (0.039)	5.801	0.055	
		T	796 (0.777)	808 (0.789)			
		C	228 (0.223)	216 (0.211)	0.414	0.520	0.933 (0.756–1.152)
rs7757931	*UBD*	CC	436 (0.852)	423 (0.826)			
		CA	74 (0.144)	82 (0.160)			
		AA	2 (0.004)	7 (0.014)	3.385	0.184	
		C	946 (0.924)	928 (0.906)			
		A	78 (0.076)	96 (0.094)	2.035	0.154	0.797 (0.583–1.089)

Bold values are statistically significant after the Bonferroni correction. CI: confidence interval; dbSNP: database of single nucleotide polymorphisms; OR: odds ratio; PD: Parkinson’s disease.

^a^Gene symbol is approved by HUGO Gene Nomenclature Committee.

^b^Genotypic or allelic frequencies are shown in parentheses.

**Table 2:** Haplotype analysis of PD-related genes comparing between PD patients and matched controls.

Gene^a^	dbSNP ID	Haplotype	Patient (%)	Control (%)	χ^2^ value	*P* value	OR (95% CI)
*ATP13A4*	rs6788448-rs35424709	T-T	58.0	55.8	0.540	0.462	1.068 (0.896–1.274)
		C-T	36.4	38.9	1.779	0.182	0.885 (0.740–1.059)
		C-A	5.3	4.0	1.816	0.178	1.331 (0.877–2.019)
		T-A	0.3	1.3	—	—	—
*FGF20*	rs1721100-rs1989754	G-G	47.3	47.8	0.049	0.824	0.980 (0.823–1.168)
		C-C	47.7	46.9	0.129	0.720	1.033 (0.867–1.230)
		G-C	3.1	3.4	0.146	0.703	0.909 (0.557–1.484)
		C-G	1.9	1.9	—	—	—
*LRRK2*	rs33949390-rs34410987	G-C	96.6	98.3	0.000	1.000	—
		C-C	2.5	1.5	—	—	—
		G-T	0.8	0.1	—	—	—
		C-T	0.1	0.1	—	—	—
*PITX3*	rs3758549-rs4919621	C-T	60.3	59.4	0.160	0.689	1.037 (0.869–1.237)
		C-A	24.7	22.4	1.571	0.210	1.140 (0.929–1.398)
		T-T	15.0	18.2	3.819	0.051	0.793 (0.627–1.001)
		T-A	0.0	0.0	—	—	—
*UBD*	rs2076485-rs7757931	T-C	70.1	70.7	0.439	0.508	0.937 (0.774–1.135)
		C-C	22.3	20.0	1.299	0.254	1.132 (0.915–1.400)
		T-A	7.6	8.2	0.370	0.543	0.905 (0.657–1.248)
		C-A	0.0	1.1	—	—	—

Haplotype with frequency less than 0.03 is not included in analysis. CI: confidence interval; dbSNP: database of single nucleotide polymorphisms; OR: odds ratio; PD: Parkinson’s disease.

^a^Gene symbol is approved by HUGO Gene Nomenclature Committee.
