# Supplementary material for: Systematic analysis of genetic variants in Han Chinese patients with sporadic Parkinson’s disease
Source: Sci Rep. 2016 Sep 22;6:33850. doi: 10.1038/srep33850 (PMC5032117; doi:10.1038/srep33850)
Supplement: Supplementary Information [file srep33850-s1.pdf]

Supplementary information

Systematic analysis of genetic variants in Han Chinese patients with sporadic Parkinson's disease

Lamei Yuan[1,2], Zhi Song[2], Xiong Deng[1], Wen Zheng[2], Yi Guo[3], Zhijian Yang[1] & Hao Deng[1,2]

[1]Center for Experimental Medicine, the Third Xiangya Hospital, Central South University, Changsha, China. [2]Department of Neurology, the Third Xiangya Hospital, Central South University, Changsha, China. [3]Department of Medical Information, Information Security and Big Data Research Institute, Central South University, Changsha, China.

Address correspondence to:

Hao Deng, M.D., Ph.D.

Professor of Center for Experimental Medicine and Professor of Neurology

Deputy Director of Center for Experimental Medicine

The Third Xiangya Hospital, Central South University

138 Tongzipo Road, Changsha, Hunan 410013, P.R. China

Tel: 86-731-88618372

Fax: 86-731-88618339

Email: hdeng008@yahoo.com

Supplementary Table

dbSNP ID	Prediction[a]	Forward primer sequence[b] (5'→3')	Reverse primer sequence[b] (5'→3')	Product size	Extending primer sequence[b] (5'→3')
rs60003608	D/Pos/DC	acgttggatgTGGACGTCTGGAAAGCAAAC	acgttggatgTTGAACTCTCAACCTGCCTC	101 bp	GGAAAGCAAACCGGATGA
rs6788448	T/P/Po	acgttggatgCCTCCTTGATGAGCAGTTTC	acgttggatgTTGTGACAGAGTGATCTGTG	105 bp	GTGATTTCTTTCTATAGGAGGTTAAT
rs35424709	D/P/Po	acgttggatgTTGTAGGCCAGTGCTATGAC	acgttggatgAACTGCGTACTAACCCTGTG	108 bp	TGTGTCGTGTAAATCTGAAG
rs6350	T/Pos/DC	acgttggatgAGGTGGAGCTCATCCTTGTC	acgttggatgGAAAGTCGATCTTCTTGCCC	126 bp	ccccaTTGTCAAGGAGCAGAA
rs13414223	T/Pos/Po	acgttggatgGTTTCTCAACTCATTGGATGC	acgttggatgTACCTCTAGTGTGTTTGCCG	94 bp	AGAAATATATTTGGAAGTAATACATAAT
rs10935014	T/Pos/DC	acgttggatgGTTGGAGGAGTCTTCTTGAG	acgttggatgGTTCTTCTCTAGGAGCTCAG	114 bp	ATTCTCTAGGCTTTCTTAGAA
rs79953286	T/Pos/Po	acgttggatgTAAAAAGGGTGATTGTGAC	acgttggatgACCTCCAGTTGGAAAGTAGG	126 bp	agGGGTGATTGTGACAAAACTT
rs2227851	D/P/DC	acgttggatgCCATCAAGAAGGAGACCAAG	acgttggatgAAGGGACCATGCAGTTAAGG	102 bp	gcAGGAGACCAAGGTTCTCAAG
rs34086109	T/Pos/DC	acgttggatgTTCCTCTCCCAGACTCTCAC	acgttggatgGAGGAGCGGAGTAGAGAAC	111 bp	ccCCGGCATCCCGCCCA
rs1721100	-	acgttggatgGTGCGATAGTGACATTATGG	acgttggatgATTCTTTCCAAATCCAGTC	138 bp	TCCCATCATAAAATAATGACCCAAG
rs1989754	-	acgttggatgCCACCCATATCTTCTTTCGC	acgttggatgTGCCTAGTTCTTCAGCCAAG	112 bp	CTTTGGAAATTGTGCAAC
rs34322892	D/Pos/DC	acgttggatgTCATTGTTCAGAGGACCATC	acgttggatgCCTGGCTTCATTGTGAAACG	108 bp	ctttCCGGCAGATCCACCC
rs34845648	T/Pos/DC	acgttggatgAAAGCTGAAGAGGAG	acgttggatgTATCAGTGATCACT	127 bp	CTACCAGCCAAAGTGC

		ACTCG	GGCCTC		
rs11538692	T/Pos/DC	acgttggatgACATCCGGCATTGTTAGCTC	acgttggatgTCAGGACGCACATCAATAGC	107 bp	AGCTCTGCTCAGCGT
rs77570025	T/B/DC	acgttggatgCAGTGGATCGTTACATAGCC	acgttggatgCACACTCTGGCATTCTCTAC	129 bp	cctccCACCACTGTAATCTTGATGA
rs2924835	D/B/Po	acgttggatgGGGTTTTGGTTGCTTAGGCG	acgttggatgAGCTCTCGGGCTTTTAAGAC	107 bp	TGGCCTGGAGAAGGATTCTG
rs3752321	D/Pos/DC	acgttggatgCCAGGGAGTTCGACATTTTC	acgttggatgGTGTGACAGTCATTCCACAG	100 bp	GACATTTTCTACCAGTCCTAC
rs3764740	D/B/Po	acgttggatgCAGCAAGTTCTTCCAGTGTG	acgttggatgTCATGCTCAGACCTGTCGG	94 bp	ggaggGGACTCAGACATGCTACATA
rs33949390	D/P/DC	acgttggatgGTTGTCCAAAACACCCTAAG	acgttggatgCTATTGGCAAAGCAATCTGG	138 bp	AAGAAATTTTTCCACATCTCTA
rs34410987	T/B/DC	acgttggatgGCCAGTCTCCTAAAAGGAAG	acgttggatgTCACGAGATCCACTATTCAG	126 bp	GTGAGAAAGAGAGCAGTC
rs34594498	D/Pos/DC	acgttggatgAGCTCATAGGGAAGTGATGC	acgttggatgAAACATGGCCCACTGCTTAC	121 bp	ttgtAAGTTTTCCAGGCATCTG
rs74942016	D/Pos/Po	acgttggatgCATAACCGGCATCTGCATCG	acgttggatgAAGCTGTCTCTGCATGAAGG	120 bp	gccgTGTGGTCGGCATCATGTGT
rs61744200	T/P/DC	acgttggatgTGTTCTAGCTACAGCTTCCC	acgttggatgGGGCTAGAATATGTTCACCC	99 bp	caAGAGACCATCTGCCC
rs11570680	T/B/DC	acgttggatgGCTTCGACTGTGCCATAGTG	acgttggatgGTCCTGGGCTCACCGACAT	105 bp	ttaTGCTGACCCACGGGGCCAAC
rs3758549	-	acgttggatgCCCCTTGCAAGAGATGATAG	acgttggatgCTCCTAGGAGAAAACCCATC	108 bp	TACCTGGAAGTGAGAATG
rs4919621	-	acgttggatgACTCTGACATGTGCTGATCC	acgttggatgTGTTCTTTGGCACACAGTGG	117 bp	ggCTACTTCCTGCCTCTATC
rs2254562	D/B/Po	acgttggatgCACCTTCCTTAGATCCAAGC	acgttggatgATTGGGCTTCTTATTTGAC	120 bp	CCAAGCAAATTTACTATTATTTGT

rs375681722	D/P/DC	acgttggatgTTGCTTAGCAGGAAGTGGTC	acgttggatgTGTCCTCGACAACAATGTGG	114 bp	AGGTGCGCATTGTC
rs538881762	D/P/DC	acgttggatgTCTGGGCAGGATTCATATTC	acgttggatgCCTTGCCTACAGGTCTAAAG	109 bp	AGGATTCATATTCATAACCCA
TENM4 p.T1367N	T/P/DC	acgttggatgTACAGTGGACAAGTTTGGGC	acgttggatgTGAGATCATTAGAGCCGAGC	104 bp	tATCGATGCGTCTGATCATG
rs2076485	D/P/Po	acgttggatgTAAGACCAAGGTTCCTGTGC	acgttggatgGTAAGGTGGATGGTCTTCTC	114 bp	gggtATGGTCTTCTCTTTGTCA
rs7757931	D/B/Po	acgttggatgTAAGCTTTTTGACCCCTGCC	acgttggatgATGGCAGATTACGGCATCAG	107 bp	TGGTCACCCTCCAATA
rs35693565	T/P/DC	acgttggatgACCCCTCAAGGCTACATATC	acgttggatgCCACATTTCGTGAGATCCAG	95 bp	CCTTCTTCCCTCCGG
rs62444122	T/Pos/Po	acgttggatgTTGCTTACCTTAAGCTGTCC	acgttggatgGCCTGGGATGTTTTTACTGC	113 bp	GTCCAATTTCTTTAAATTTATAAACTT
rs199910950	D/P/DC	acgttggatgCCATTCCTTCCTTAATACGG	acgttggatgTTATGTGGTATTTTGTAGC	99 bp	tttgGTAGCATTTATTACTGGCTT

Supplementary Table S1. Primer sequences designed by Sequenom Assay Design 3.1 software (Sequenom, San Diego, CA, USA). B: benign; D: damaging; DC: disease causing; P: probably damaging; Po: polymorphism; Pos: possibly damaging; T: tolerated. [a] Sorting Intolerant from Tolerant/Polymorphism Phenotyping version 2/MutationTaster prediction. [b] The lowercase letters in the primer sequences are 5'-end tags (forward and reverse primers) or nonhomologous sequences (extending primers) to increase the molecular weights.
